# Prediction of concurrent chemoradiotherapy outcome in advanced oropharyngeal cancer

**DOI:** 10.3892/ijo.2014.2504

**Published:** 2014-06-18

**Authors:** MASAHIRO HASEGAWA, HIROYUKI MAEDA, ZEYI DENG, ASANORI KIYUNA, AKIRA GANAHA, YUKASHI YAMASHITA, SEN MATAYOSHI, SHINYA AGENA, TAKAFUMI TOITA, TAKAYUKI UEHARA, MIKIO SUZUKI

**Affiliations:** 1 Department of Otorhinolaryngology, Head and Neck Surgery, Graduate School of Medicine, University of the Ryukyus, Okinawa 903-0215, Japan; 2 Department of Radiology, Graduate School of Medicine, University of the Ryukyus, Okinawa 903-0215, Japan; 3 Department of Otorhinolaryngology, Head and Neck Surgery, Zhujiang Hospital, Southern Medical University, Guangzhou, P.R. China

**Keywords:** oropharyngeal cancer, chemoradiotherapy, planned neck dissection, human papillomavirus

## Abstract

The aim of this study was to investigate human papillomavirus (HPV) infection as a predictor of concurrent chemoradiotherapy (CCRT) response and indicator of planned neck dissection (PND) for patients with advanced oropharyngeal squamous cell carcinoma (OPSCC; stage III/IV). Overall, 39 OPSCC patients (32 men, 7 women; median age 61 years, range 39–79 years) were enrolled. The primary lesion and whole neck were irradiated up to 50.4 Gy, and subsequently the primary site and metastatic lymph nodes were boosted with a further 16.2 Gy. Although several chemotherapy regimens were employed, 82.1% of OPSCC patients received the combination of nedaplatin and 5-fluorouracil. HPV-related OPSCC (16 cases) was defined as both HPV DNA-positive status by polymerase chain reaction and p16^INK4a^ overexpression by immunohistochemistry. Patients with N2 and N3 disease received PND 2–3 months after CCRT completion. Compared to non-responders, CCRT responders showed significantly lower nodal stage (N0 to N2b) and HPV-positive status in univariate analysis. Patients with HPV-related OPSCC had longer time to treatment failure (TTF) than those with HPV-unrelated OPSCC (p=0.040). Three-year TTF was 81.3 and 47.8% in the HPV-related and HPV-unrelated groups, respectively. There were also significant differences in disease-free survival (DFS) between the two OPSCC patient groups (p=0.042). Three-year DFS was 93.8 and 66.7% in patients with HPV-related and HPV-unrelated OPSCC, respectively. Multivariate logistic analysis showed a lower risk of TTF event occurrence in HPV-related OPSCC (p=0.041) than in HPV-unrelated OPSCC. Thus, HPV testing in addition to nodal stage was useful for predicting CCRT response, especially in advanced OPSCC. Because patients who received PND showed moderate locoregional control, PND is an effective surgical procedure for controlling neck lesions in patients with advanced HPV-unrelated disease.

## Introduction

Surgical treatment for oropharyngeal squamous cell carcinoma (OPSCC) sometimes leads to prolonged dysphagia and aspiration pneumonia as complications of surgery. As an alternative to primary surgery, radiotherapy (RT) combined with chemotherapy, especially concurrent chemoradiotherapy (CCRT), has become a widely accepted treatment for organ preservation in selected patients with advanced head and neck cancer, including OPSCC (1–4). However, cervical lymph node metastasis is usually less responsive to CCRT than the primary lesion, and it remains viable in 26% of patients with a clinical complete response (CR) even after CCRT (5) and in 25.9% of patients following planned neck dissection (PND) based on pretreatment N2a or greater neck disease or post-treatment residual disease (6). Thus, accurate detection of residual primary or neck lesions is important in the follow-up of OPSCC patients treated with CCRT. A clinical CR of neck disease after radiotherapy does not reliably determine the pathologic status of the neck (6,7), but Goenka *et al* demonstrated that positron emission tomography (PET) and computed tomography (CT) findings can indicate the need for neck dissection in OPSCC patients after CCRT (8).

Salvage neck dissection after CCRT is in certain cases difficult to perform because of severe scarring caused by the CCRT and significant complications after surgery (9). PND has conventionally been performed 6–10 weeks after finishing CCRT regardless of the nodal response after definitive radiotherapy (RT) in patients with advanced head and neck squamous cell carcinoma (HNSCC), especially those with N2 or N3 status (10). There have been contradictory reports on the early diagnosis of residual lesions and optimal surgical management of neck metastatic lesions after CCRT. Because dissected neck tissue at PND does not usually scar severely, in contrast to salvage neck dissection, PND is feasible, safe, and correlates with clinical outcome in HNSCC patients (6,11,12). However, other reports indicate that that surgery after CCRT does not further reduce the risk of a regional occurrence or distant failure or improve survival after a clinical/radiographic neck CR, and in fact it increases morbidity (13–15).

The two most important risk factors for the development of HNSCC are heavy smoking and extensive alcohol consumption (16). Recently, a strong correlation between human papillomavirus (HPV) and HNSCC has been established in OPSCC, particularly tonsillar carcinoma, with HPV DNA present in up to 70% of the patients studied (17–19). Furthermore, many studies have demonstrated that HPV-positive OPSCC patients have a better prognosis than HPV-negative OPSCC patients (18,20). According to National Comprehensive Cancer Network (NCCN) guidelines version 2, 2013, although tumor HPV testing for OPSCC is recommended, the test results should not change management decisions except in the context of a clinical trial. There have been few reports of the efficacy of HPV testing with regard to PND for OPSCC patients with N2/N3 status (21,22). Therefore, this study aimed to clarify HPV infection as a predictor of CCRT response and an indication for PND in patients with advanced OPSCC.

## Materials and methods

Between 2006 and 2012, 53 patients were diagnosed with OPSCC by pathologic examination of biopsy samples at the Department of Otorhinolaryngology, Head and Neck Surgery at the University of the Ryukyus. Classification of the TNM stage was performed according to the American Joint Committee on Cancer (AJCC; 7th Edition, 2009), and tissue samples were differentiated into grades according to the World Health Organization International Histological Classification of Tumors.

Following approval of the research protocol by the Institutional Review Board of the University of the Ryukyus, informed consent was obtained from all OPSCC patients before being enrolled into this study.

### Patient selection and CCRT treatment protocol

Eligibility criteria were as follows: the presence of untreated, pathologically confirmed primary OPSCC without distant metastases (M0), advanced clinical AJCC stage (stage III, IVA or IVB), age ≥18 years, and CCRT as a principal treatment with curative intent. Locoregional response to CCRT was determined by clinical and radiological (CT, magnetic resonance imaging, or PET-CT) examination as well as surgical biopsy in cases where local recurrence was strongly suspected.

**Figure 1:**
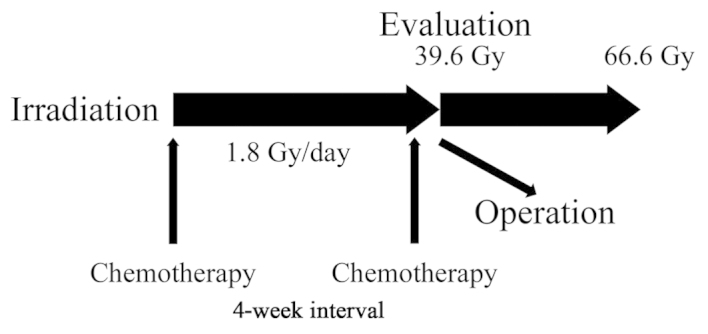
Representative treatment scheme. The main chemotherapy regimen was a combination of nedaplatin and 5-fluorouracil (FN regimen), which was, in principle, administered twice with a 4-week interval. A radiographic response of the primary lesion was determined at 39.6 Gy irradiation in all patients to determine whether concurrent chemoradiotherapy would be continued or whether surgery was warranted.

A representative treatment scheme is shown in Fig. 1. The details of CCRT were obtained from medical charts and radiotherapy records. All patients had CT-assisted 3-dimensional radiation treatment planning in the treatment position with mask immobilization. The oropharynx and upper neck were treated through bilaterally opposed fields. The bilateral lower neck down to the supraclavicular fossa was treated with separate anteroposterior-posteroanterior portals that allowed shielding of the larynx and spinal cord. The primary lesion and whole neck including bilateral neck lymph nodes were irradiated with 1.8 Gy per day, up to 50.4 Gy. The primary site and metastatic lymph nodes were subsequently boosted with a further 16.2 Gy in 9 fractions. The accumulated dose to the gross primary tumor and metastatic neck lymph nodes was 66.6 Gy at 1.8 Gy/day, 5 times a week, in 37 fractions (once daily fractionation).

Several chemotherapy regimens were employed in this study. A summary of chemotherapy regimens is shown in Table I. The main chemotherapy regimen was a combination of nedaplatin (CDGP) and 5-fluorouracil (5-FU) (FN regimen). In the FN regimen, CDGP was administered at a dose of 90 mg/m^2^ on day 1 and 5-FU was administered continuously at a dose of 800 mg/m^2^ on days 2–6; the regimen was, in principle, given twice with a 4-week interval. The combination of docetaxel, CDGP, and 5-FU (TPF regimen) consisted of continuous administration of 5-FU (600 mg/m^2^) on days 1–4 and administration of CDGP (60 mg/m^2^) and docetaxel (60 mg/m^2^) on day 2; the weekly docetaxel regimen (weekly TXT) consisted of administration of docetaxel 10 mg, 5 times. The chemotherapy regimen administered was determined according to the patient’s general condition and any complications and at the discretion of the radiologist and surgeon.

The radiological response of the primary lesion was determined at 39.6 Gy by CT, according to the new Response Evaluation Criteria in Solid Tumours (revised RECIST guidelines, version 1.1) (23). If the primary lesion showed a partial response (≥30% reduction in size), CCRT continued as per the protocol. When the primary tumor failed to show a partial response regardless of the neck lymph node response, patients underwent curative surgery for the primary lesion combined with neck dissection. Patients who had N2 and N3 lesions were planned to receive neck dissection 2–3 months after CCRT (PND). The decision to perform comprehensive or selective neck dissection was made by the surgeon.

Every lymph node and extranodal deposit at PND was classified pathologically as either having no disease, treated disease with no viable cells, or residual viable cells. A pathologic CR in the neck was defined as the absence of viable tumor in any of the cervical lymph nodes or cervical soft tissues. Primary site response to CCRT was assessed with a combination of clinical and imaging examinations. All patients underwent a pan-endoscopy of the head and neck region at each visit during the observation period. In addition, biopsy of the primary site and fine needle aspiration of the neck lymph node were used in patients with suspected recurrence.

### Clinicopathologic parameters and follow-up

Clinicopathologic parameters and treatment outcome for each patient were recorded at scheduled intervals during the observation period. Clinical parameters were statistically compared between subjects using Fisher’s exact test. The status of each patient, including information about recurrence and metastasis, was reported at least every 4–6 weeks for the first year, every 2–3 months from 2–5 years, and thereafter every 6 months.

### HPV infection in oropharyngeal lesions

All tissue samples of primary lesions were analyzed by both polymerase chain reaction (PCR) and p16^INK4a^ immunohistochemistry. HPV-related OPSCC was defined as both HPV DNA-positive status and p16^INK4a^ overexpression (positive cells in >40% of the tumor).

#### DNA extraction and PCR for detection of high- and low-risk HPV DNA

DNA extraction and PCR methods for detection of HPV DNA have been described previously (19,20). In brief, the obtained tissue samples were snap-frozen in liquid nitrogen during biopsy. A Gentra purification tissue kit (Qiagen, Germantown, MD, USA) was used to isolate DNA from the samples. The presence and integrity of DNA in all samples was verified by PCR β-globin gene amplification using the primers PC04 and GH20.35. Water (negative control) and the DNA of HPV-16-positive CaSki cells (positive control) were included in each amplification series. The presence of high- and low-risk HPV DNA was analyzed by PCR using the general consensus primer sets GP5^+^/GP6^+^ and MY09/11 (24,25). DNA samples that were negative in the GP5^+^/GP6^+^ or MY09/11 PCR were re-amplified in an (auto-) nested PCR using the GP5^+^/GP6^+^ primer pair as previously described (26) to increase the sensitivity of HPV detection. Obtained sequences were aligned and compared with those of known HPV types in the GenBank database using the BLAST program.

#### Immunohistochemistry for p16^INK4a^

Serial sections (4-μm thick) of formalin-fixed paraffin-embedded tumor samples were deparaffinized in xylene and subsequently rehydrated through a graded alcohol series. Epitope retrieval was performed by heating at 95–99°C for 10 min in Tris-EDTA buffer (pH 9.0). Endogenous peroxidase activity was quenched by incubating the sections in 3% hydrogen peroxide plus 15 mM sodium azide for 5 min. The sections were subsequently incubated overnight at 4°C with primary monoclonal mouse anti-p16^INK4a^ antibody (MTM Laboratories AG, Heidelberg, Germany). After extensive washing in phosphate-buffered saline, the slides were incubated for 30 min at room temperature with a horseradish peroxidase-conjugated goat anti-mouse secondary antibody (MTM Laboratories). Immunolabeling was visualized by incubation in 3-3′-diaminobenzidine for 10 min. Stained slides were counterstained with hematoxylin. The scoring criteria for p16^INK4a^ immunoreactivity (p16^INK4a^ expression) were defined for this study based on previous scoring methods (27,28): 0 (no staining), 1 (1–10% of tumor cells positive), 2 (11–40% positive), 3 (40–70% positive) and 4 (>70% positive). The term ‘p16^INK4a^ overexpression’ is defined as a score of 3 or 4.

### Survival estimation

Response to CCRT was classified into 7 categories: 1, progressive disease (PD) or stable disease (SD) at 39.6 Gy in CCRT; 2, residual lesion in lymph node or primary lesion at PND; 3, locoregional recurrence after CCRT during observation period with or without distant metastasis; 4, distant metastasis after treatment without logoregional recurrence; 5, OPSCC-unrelated death without recurrence or distant metastasis; 6, no residual locoregional lesions at PND; and 7, no recurrence and no distant metastasis. The final prognosis of patients was judged in December 2013. CCRT responders were classified as having a CCRT response of 6 or 7 during the observation period.

Time to treatment failure (TTF), disease-free survival (DFS), and overall survival (OS) were investigated as survival indicators. Survival curves were estimated according to the Kaplan-Meier method, and survival distributions were compared using the log-rank test. TTF was defined as the time from the start of CCRT to the appearance of CCRT response events 1–5. Those who were alive without achieving CCRT response events 1–5 were censored in December 2013, and those who died from any other cause unrelated to the disease were censored at the date of death. DFS was defined as the time from the end of treatment (i.e., CCRT, PND, or surgical resection because of poor response to CCRT) to achieving CCRT response 3 or 4 or to December 2013. DFS denotes the probability of remaining free of disease after CCRT. OS was defined as the time from the start of treatment to death from any cause (both related and unrelated to OPSCC) or December 2013.

All factors were considered to be categorical variables for survival estimation. All tests were two-sided and p-values <0.05 were considered statistically significant. The multivariate prognostic significance of tumor variables on TTF and DFS were assessed using logistic analysis with a forward selection procedure and a significance level determined by the Wald test for a covariate to remain in the model specified at p<0.10. Analyses were performed using the SPSS statistical package (SPSS for Windows version 19.0; SPSS, Inc., Chicago, IL, USA).

## Results

### Clinicopathologic parameters

A total of 39 patients (32 men, 7 women; median age 61, range 39–79 years; Table II) with OPSCC met the inclusion criteria. Of these 39 patients, 28 had lesions in the lateral portion of the oropharynx and 11 had lesions in other parts of the oropharynx. The clinical characteristics including T and N stage disease status are shown in Table II. Ten patients were classified as stage III, 22 as IVA, and 7 as IVB. The follow-up period, excluding those patients who died during this time, was 18–82 months, with a median of 50 months for patients whose data were censored.

**Figure 2:**
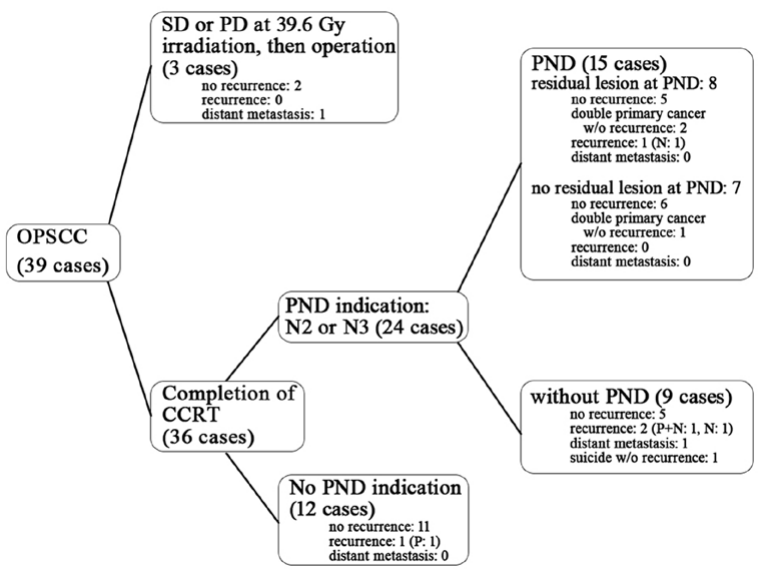
Survival events and treatment. Three patients failed to show a partial response at 39.6 Gy and therefore underwent curative surgery for the primary and neck lesions. All other patients proceeded to the concurrent chemoradiotherapy protocol. CCRT, concurrent chemoradiotherapy; OPSCC, oropharyngeal squamous cell carcinoma; N, nodal recurrence; P, oropharyngeal recurrence; N+P, nodal and oropharyngeal recurrence; PD, progressive disease; PND, planned neck dissection; SD, stable disease; w/o, without.

Three patients failed to reach PR at 39.6 Gy and underwent curative surgery for the primary and neck lesions. All other patients proceeded to the CCRT protocol. Fifteen of the 24 patients who were indicated for PND underwent the procedure; the remaining 9 patients did not receive PND because of refusal, general physical condition, or surgeon’s decision. Information regarding survival events is summarized in Fig. 2. One PND case showed a residual tonsillar lesion without residual neck lesion at PND. Overall, total primary oropharyngeal control in response to CCRT failed in 6 (15.3%) of the 39 OPSCC patients (3 with SD or PD at 39.6 Gy irradiation, the above-mentioned case with the local residual tonsillar lesion, and 2 with primary recurrence during post-CCRT observation). Total neck control in response to CCRT failed in 13 (33.3%) of the 39 OPSCC patients (3 with SD or PD at 39.6 Gy irradiation, 8 with residual lesions at PND, 1 with neck recurrence without a primary lesion, and 1 with neck and oropharyngeal recurrence). One patient who had no recurrence and no metastatic lesions at the final visit was lost to follow-up after 18 months.

The number of patients who received each chemotherapy regimen is shown in Table I. A total of 32 (82.1%) of the 39 participants received the FN regimen. Two of these patients could not receive the 2nd chemotherapy cycle due to side effects, but were able to continue irradiation. One patient who received 4 cycles of the TXT regimen could not continue chemotherapy or radiation therapy due to side effects; irradiation was stopped at 52.2 Gy. The median overall treatment time was 57 days (range, 49–77 days) for 35 patients who completed the planned radiotherapy.

CCRT responders had significantly lower N stage (N0 to N2b) and were positive for HPV infection in univariate analysis compared to CCRT non-responders. The other parameters of gender, age, tumor location, histologic differentiation, T stage, smoking, and alcohol consumption did not impact on CCRT response in OPSCC patients. Multiple regression logistic analysis showed that CCRT responders had a significantly low N stage (p=0.021) but were not HPV-positive (p=0.128). Only 1 patient showed recurrence postoperatively among the 15 patients who received PND, whereas 3 of 9 patients who were indicated for PND, but did not undergo the procedure, had recurrence or metastasis (Fisher’s exact test, p=0.1304).

Patients underwent temporary tracheostomy after PND, but none required prolonged tracheostomy except for a patient who died due to recurrence. Further, as of December 2013, 12 of the 15 patients who underwent PND were able to eat normally whereas the remaining 3 could only eat soft food. No patients required tube feeding. There were no wound healing complications.

### PCR results

HPV DNA was detected by PCR in 22 of the 39 (56.4%) specimens. Among the HPV-positive OPSCC samples, 16 (72.7%) were infected with HPV-16 and the others were infected with other high-risk types (3 with HPV-33, 1 with HPV-35, and 2 with HPV-58). No patients were infected with low-risk or multiple HPV types.

### p16^INK4a^ expression and correlation with HPV status

**Figure 3:**
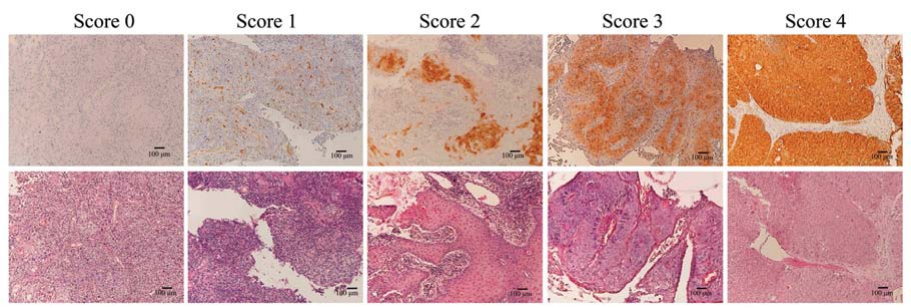
Examples of p16^INK4a^ immunoreactivity scores. The scoring criteria for p16^INK4a^ immunoreactivity were 0 (no staining), 1 (1–10% of tumor cells positive), 2 (11–40% positive), 3 (40–70% positive), and 4 (>70% positive). p16^INK4a^ overexpression was defined as a score of 3 or 4.

Fig. 3 shows examples of the scoring of p16^INK4a^ expression. The p16^INK4a^ overexpression (score 3 or 4) was observed in 16 of the 39 OPSCC patients. Although all patients with p16^INK4a^ overexpression were also HPV DNA-positive, 16 of 22 patients who had HPV DNA showed p16^INK4a^ overexpression. Therefore, 16 OPSCC patients displayed both HPV DNA-positive status and p16^INK4a^ overexpression.

### Prognosis

**Figure 4:**
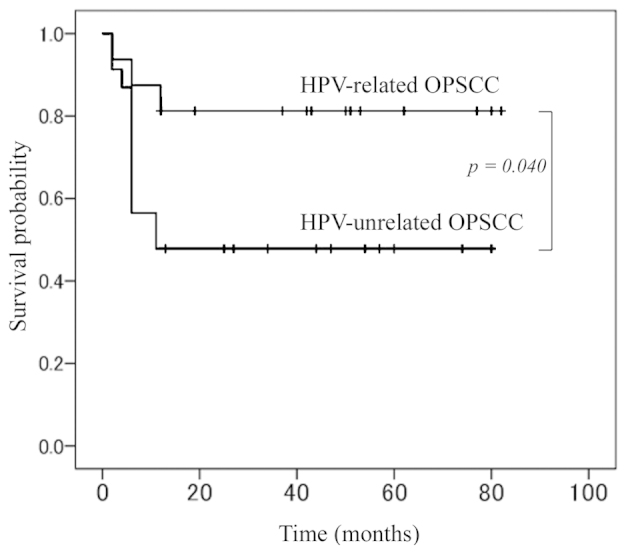
Kaplan-Meier analysis of time to treatment failure in HPV-related and HPV-unrelated oropharyngeal squamous cell carcinoma (OPSCC). Patients with HPV-related OPSCC had longer time to treatment failure (TTF) than those with HPV-unrelated OPSCC (p=0.04). The 3-year probability of TTF was 81.3 and 47.8% in the HPV-related and HPV-unrelated OPSCC groups, respectively.

**Figure 5:**
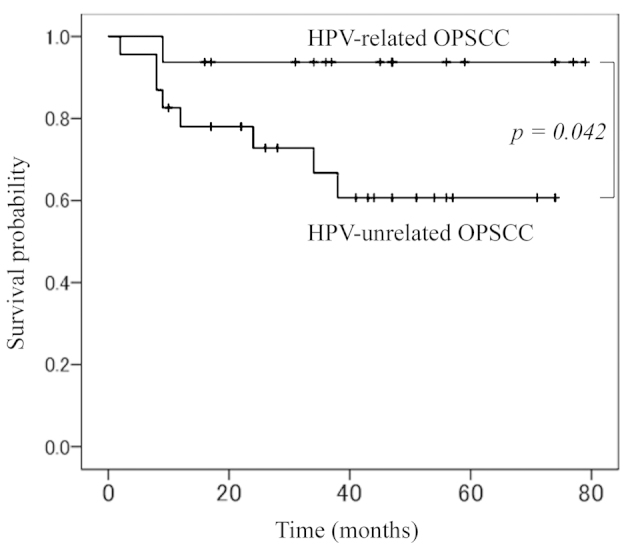
Kaplan-Meier analysis of disease-free survival in HPV-related and HPV-unrelated oropharyngeal squamous cell carcinoma (OPSCC). There was a significant difference in disease-free survival (DFS) between the two groups (p=0.042). The 3-year probability of DFS was 93.8 and 66.7% in the HPV-related and HPV-unrelated OPSCC groups, respectively.

In Kaplan-Meier analysis, patients with HPV-related OPSCC had longer TTF than those with HPV-unrelated OPSCC (p=0.040, Fig. 4). The 3-year probability of TTF was 81.3 and 47.8% in the HPV-related and -unrelated OPSCC patients, respectively. There was also a significant difference in DFS between the two groups (p=0.042, Fig. 5). The 3-year probability of DFS was 93.8 and 66.7% in the HPV-related and in HPV-unrelated OPSCC patients, respectively. However, there was no significant difference in OS between the two groups (p=0.091, Kaplan-Meier curve is not shown). The 3-year rate of OS was 100 and 81.5% in the two groups, respectively. The 3-year OS in all 39 OPSCC patients was 88.9%.

Univariate analysis carried out to determine the effect of smoking, alcohol consumption, tumor location, T stage, N stage, histologic differentiation, and HPV infection on TTF and DFS in 39 OPSCC patients (Table III) revealed that nodal stage and HPV presence were significantly related to TTF (p=0.010 and p=0.040, respectively) and DFS (p=0.006 and p=0.042, respectively). Multivariate logistic analysis was carried out to identify the effect of independent risk factors on TTF and DFS in OPSCC. The final model showed that there was a higher risk for N2c or N3 disease in relation to TTF (p=0.021) and DFS (p=0.013) compared with N0, N1, N2a, or N2b disease (Table III).

Univariate analysis performed to determine the effect of smoking, alcohol consumption, tumor location, T stage, N stage, histologic differentiation, and HPV infection on TTF in the 24 OPSCC patients indicated for PND (e.g., patients treated with CCRT and with initial nodal N2 or N3) (Table IV) revealed HPV presence was significantly related to TTF (p=0.029). In multivariate logistic analysis to identify independent risk factors with an effect on TTF in these 24 patients, the final model showed that there was a lower risk for HPV-related OPSCC in relation to TTF (p=0.041) compared with HPV-unrelated OPSCC (Table IV).

## Discussion

The increasing prominence of multimodality therapy for patients with advanced head and neck cancer reflects its high survival and functional preservation rates. CCRT is emerging as one of the most successful treatments for patients with advanced OPSCC in terms of organ and functional preservation and survival. The primary and nodal metastasis control rate in the present study was 84.7 and 66.7% in stage III or IV OPSCC patients (n=39), respectively. Locoregional residual or recurrent lesions were successfully salvaged and 3-year OS in all patients was relatively good (88.9%), which is in agreement with the findings of recent reports (8,15). In addition, the control rate of primary lesions in the present study was better than that of neck lymph nodes, which is in line with the results of a previous study (29). Of the 12 patients with N0 or N1 disease, only 1 showed recurrence. By contrast, of the 24 patients with N2 or N3 disease, 10 (41.7%) showed residual or recurrent lesions in the lymph nodes. From these findings, it appears that the indications for PND applied in the present study were suitable for the selection of patients who needed PND. Since PND provides good control of nodal metastasis in OPSCC, in some cases no residual lesions were observed in PND specimens. Approximately 60% of patients with N2 or N3 disease in the present study still do not need neck dissection for control of lymph node metastasis seen on pathologic examination of the dissected tissue. With regard to complications of PND, 4 (7.0%) of 57 patients with supraglottic carcinoma who received PND after RT displayed severe complications (e.g., skin breakdown or carotid artery rupture), due to PND (30). Davidson *et al* reported that 15 (36.6%) of 41 patients undergoing neck dissection after radiation or CCRT (≥70 Gy) showed significant wound complications (31). However, Brizel *et al* reported that modified neck dissection still appears to confer a DFS and OS advantage with acceptably low morbidity in patients with N2/N3 neck disease undergoing CCRT (5). Hillel *et al* reported similar complication rates and equivalent regional control rates in patients undergoing either comprehensive or selective neck dissection (29). We performed selective neck dissections for most patients and reserved more radical surgery for patients with extensive residual neck disease, in accordance with a previous study (12). In the present study, there were no serious complications such as carotid artery rupture or reoperation to manage a complication, although patients had a temporary tracheostomy for laryngeal edema. These findings suggest that severe complications do not occur often in PND and that it can be performed safely with an acceptable incidence of late complications of the airways and dysphagia.

The findings of several studies suggest that p16^INK4a^ expression can be used as a surrogate marker of HPV infection in HNSCC (27,32). Klaes *et al* defined p16^INK4a^ staining as negative, sporadic, focal, or diffuse and found a significant correlation between the presence of high-risk HPV and strong diffuse p16^INK4a^ expression (27). However, there are also several contradictory reports on the value of p16^INK4a^ as a biomarker. Smith *et al* found no concordance between p16^INK4a^ expression and HPV detection in 20% of head and neck cancers (33), possibly due to transcriptionally inactive infection or an alternate pathway of p16^INK4a^ activation (34). Using the same histological criteria as Klaes *et al* (27), Hoffmann *et al* reported that 81.2% of HPV DNA-positive HNSCC patients were also p16^INK4a^ positive, compared with only 48.2% of HPV DNA-negative cases, including 3 cases with strong diffuse p16^INK4a^ staining. Moreover, no p16^INK4a^ expression could be detected in 3 of 14 HPV DNA^+^/RNA^+^ HNSCC lesions in their series. These authors concluded that p16^INK4a^ expression status alone is inadequate for identifying biological active or inactive HPV infections in HNSCC (35). Therefore, in the present study, HPV-related OPSCC was defined as both HPV DNA detected on PCR and p16^INK4a^ overexpression detected on immunohistochemistry.

Hillel *et al* reported that 6 (33.3%) of 18 HPV-positive patients had residual disease following CRT, whereas 2 (18%) of 11 HPV-negative patients had residual disease, with no significant difference (p=0.238) between the presence of a residual lesion and HPV status (29). By contrast, Shonka *et al* reported that PND for p16-negative OPSCC was significantly more likely to show viable tumor in the specimens than PND for p16-positive OPSCC, while p16-positive OPSCC was associated with more advanced neck lesions (21). In the present study, the rate of HPV-related OPSCC in the CCRT responder group was higher than in the CCRT non-responder group, which concurs with the findings of Shonka *et al* (21). However, multivariate analysis only showed a significant correlation between nodal stage and prognosis, while univariate analysis demonstrated that patients with HPV-positive status and lower nodal stage (N0, N1, N2a, or N2b) had longer TTF and DFS. These results indicate that nodal stage is the most important variable for prediction of CCRT response in advanced OPSCC. On the other hand, survival analysis based on the 24 OPSCC patients with N2 or N3 disease demonstrated longer TTF in HPV-related OPSCC in multivariate analysis. Because OPSCC patients usually show an excellent response to CCRT, early nodal stage (i.e. N0, N1, N2a, or N2b) disease was successfully treated regardless of HPV presence. However, HPV positivity is an important indicator of CCRT response in patients with advance nodal stage (i.e. N2c or N3) disease. Huang *et al* reported that HPV-related unrelated head and neck cancer with positive nodes in PND specimens had better 5-year OS, but similar local, regional, and distant control, compared with HPV-unrelated head and neck cancer (22). As mentioned above, because the primary lesions were well controlled by CCRT in the present study, the control of nodal metastasis is crucial for obtaining a fair prognosis. These findings suggest that HPV testing is important for determining whether PND is indicated.

In conclusion, HPV testing as well as nodal stage was useful for predictng the CCRT response, especially in OPSCC patients with advanced nodal stage disease. Because patients who received PND showed reasonable locoregional control, PND is an effective surgical procedure for controlling neck lesions in patients with HPV-unrelated advanced nodal stage OPSCC. Because the number of cases was limited and this investigation was retrospective, a prospective study is needed to confirm the present findings.

## Figures and Tables

**Table I:** Chemotherapy regimens.

1st time	2nd time	No. of patients
Nedaplatin + 5-FU	Nedaplatin + 5-FU	31
	Discontinuation due to side effects	1
TPF	Nedaplatin + 5-FU	3
	Discontinuation due to side effects	1
Weekly TXT i.v. (5 courses)		1
	Discontinuation due to side effects (4 courses)	1
Weekly TXT i.v. (2 courses)	Nedaplatin + 5-FU	1

5-FU, 5-fluorouracil; TPF, combination of nedaplatin, docetaxel, and 5-FU; TXT, docetaxel; i.v., intravenous.

**Table II:** Clinical characteristics of the patients.

	All patients	CCRT non-responders	CCRT responders	p-value between CCR non-responders vs. responders
No. of cases	39	15	24	
Mean age (years)	61	60.5	61.3	
Gender				1.0000
Male	32	12	20	
Female	7	3	4	
Tumor location				0.4770
Lateral	28	12	16	
Other	11	3	8	
Histologic differentiation				0.1504
Well/moderate	28	13	15	
Poor	11	2	9	
T stage				0.5145
T1, T2	20	9	11	
T3, T4	19	6	13	
N stage				0.1706
N0, N1	14	3	11	
N2, N3	25	12	13	
				0.0306
N0, N1, N2a, N2b	27	7	20	
N2c, N3	12	8	4	
Multiple primary				0.6857
Yes	8	4	4	
No	31	11	20	
Smoking (BI)				0.3178
≥800	16	8	8	
<800	23	7	16	
Alcohol consumption				1.0000
Drinker	33	13	20	
Non-drinker	6	2	4	
HPV DNA				0.5078
Positive	22	7	15	
Negative	17	8	9	
p16^INK4a^ overexpression				0.0485
Positive	16	3	13	
Negative	23	12	11	
HPV-related OPSCC				0.0485
Yes	16	3	13	
No	23	12	11	

BI, Brinkman index; CCRT, concurrent chemoradiotherapy; OPSCC, oropharyngeal squamous cell carcinoma.

**Table III:** Univariate and multivariate analysis of time to treatment failure and disease-free survival in 39 OPSCC patients.

	Time to treatment failure			Disease-free survival		
		
	Univariate analysis	Multivariate analysis		Univariate analysis	Multivariate analysis	
		
Variables	p-value	p-value	95% CI	p-value	p-value	95% CI
Smoking (Brinkman index)						
≥800 vs. <800	0.166			0.748		
Alcohol consumption						
Drinker vs. non-drinker	0.868			0.456		
Tumor location						
Lateral vs. other	0.467			0.253		
T stage						
T1, T2 vs. T3, T4	0.484			0.649		
N stage						
N0, N1, N2a, N2b vs. N2c, N3	0.010	0.021	0.040–0.766	0.006	0.013	0.024–0.651
Histological differentiation						
Well/moderate vs. poor	0.130	0.32		0.699		
HPV-related vs. HPV-unrelated	0.040	0.135		0.042	0.135	

CI, confidence interval; HPV, human papillomavirus.

**Table IV:** Univariate and multivariate analysis demonstrating the prognostic impact on time to treatment failure in 24 patients with N2 or N3 OPSCC.

	Time to treatment failure		
	
	Univariate analysis	Multivariate analysis	
		
Variables	p-value	p-value	95% CI
Smoking (Brinkman index)			
≥800 vs. <800	0.808		
Alcohol consumption			
Drinker vs. non-drinker	0.142		
Tumor location			
Lateral vs. other	0.099	0.103	
T stage			
T1, T2 vs. T3, T4	0.151		
N stage			
N2a, N2b vs. N2c, N3	0.075	0.414	
Histological differenciation			
Well/moderate vs. poor	0.388		
HPV-related vs. HPV-unrelated	0.029	0.041	1.081–47.962

CI, confidence interval; HPV, human papillomavirus.
